# Invasive *Mycobacterium* *marinum* Infections

**DOI:** 10.3201/eid0911.030192

**Published:** 2003-11

**Authors:** Timothy Lahey

**Affiliations:** * Harvard Medical School, Boston, Massachusetts, USA

**To the Editor:** *Mycobacterium marinum* infections, commonly known as fish tank granuloma, produce nodular or ulcerating skin lesions on the extremities of healthy hosts. Delay of diagnosis is common, and invasion into deeper structures such as synovia, bursae, and bone occurs in approximately one third of reported case-patients (*1*).

A 49-year-old man with diabetes, who had received kidney transplant from a living relative 8 years previously, sought treatment after 5 months of worsening swelling and tenderness of the left elbow. Of note, he had injured his left ring finger while cleaning barnacles from a piling 5 years previously and had contracted a secondary infection that never completely healed despite three courses of antimicrobial drugs and surgical debridement. Physical examination showed marked swelling, tenderness, and warmth of the left elbow, as well as of the left ring finger, which was erythematous. Sterile aspiration of the olecranon bursa showed 7,500 leukocytes (62% lymphocytes) and 141,000 erythrocytes. Results of Gram stain and routine cultures were negative. Magnetic resonance imaging of the left arm showed soft tissue edema of the olecranon bursa and the left fourth flexor digitorum longus tendon, and no osteomyelitis. Three weeks later, olecranon bursa aspirate fluid cultures incubated on chocolate agar and 7H11 plates at 31°C, as well as on algae slant, and mycobacterial growth indicator tubes incubated at 37°C grew *M.ycobacterium marinum*. The isolate was susceptible to most agents but showed intermediate susceptiblity to ciprofloxacin (MIC 2 μg/mL) and was resistant to ampicillin/clavulanate and erythromycin (MIC 8 μg/mL and 32 μg/mL, respectively). A treatment regimen of rifampin and ethambutol was begun, and the patient showed a dramatic improvement in the ensuing several weeks. The patient has completed 9 of 11.4 planned months of therapy and continues to do well, with frequent office visits.

Case reports from English language MEDLINE articles since 1966 under the subject heading *Mycobacterium marinum* were cross-referenced with articles containing the following text words: disseminated, osteomyelitis, arthritis, synovitis, and bursitis. Ten case reports were identified, and a hand search through pertinent articles’ references yielded 13 additional reports. A total of 35 cases of invasive *M. marinum* disease were then reviewed, according to patient age and sex, symptoms, source of infection, immune impairment, time to diagnosis, and type as well as duration of therapy (*2*–*24*) (Table).

**Table:** Summary of Invasive *Mycobacterium marinum* infection cases published since 1966

Ref.	Age	Sex	Symptoms	Source	Immune impairment	Dx delay (mo)	Medications	Surgery ?	Treatment duration (mo)
(*2*)	32	M	Disseminated cutaneous, larynx	Minor trauma	Systemic steroids	240	I, S	Yes	>36
(*3*)	37	M	R middle finger osteomyelitis	Fishing injury	Local steroid injection	3	Erythromycin, S	Yes	5
(*3*)	30	M	R long finger osteomyelitis	Minor trauma	None	12	Declined	No	--
(*3*)	56	M	R ring finger tenosynovitis	Minor trauma, fishing and cleaning boats	Local steroid injection	1.2	E	Yes	9
(*3*)	52	M	L index finger osteomyelitis and synovitis	None	Local steroid injection	7	Cycloserine, ethionamide, R	Yes	?
(*3*)	60	M	R ring finger synovitis and osteomyelitis	Shrimp cleaning injury	None	7	E, R	Yes	6
(*3*)	55	M	L long finger synovitis and skin ulcer	Shrimp cleaning injury	None	23	E	Yes	6
(*4*)	30	M	Disseminated skin nodules, lymphangitis	Aquarium cleaning	Systemic steroids and azathioprine for renal transplant	2	E, R, T	No	?
(*5*)	1.3	F	Disseminated cutaneous nodules	Aquarium exposure	None	2	E, I, R	No	8
(*6*)	26	M	R hand tenosynovitis	Fisherman	Local steroid injection	8	E, P, R, tetracycline	Yes	?
(*6*)	45	M	R index tenosynovitis	Saltwater fishing	Local steroid injection	1	E, R, tetracycline	Yes	?
(*6*)	22	M	R hand tenosynovitis	Fisherman	None	2	I, R, S	Yes	?
(*6*)	56	F	L hand tenosynovitis	Fishing injury	Local steroid injections	5	I, R, S	Yes	?
(*6*)	53	M	R hand and forearm synovitis, bursitis	Fisherman	Local steroid injections	78	E, I, R, S	Yes	?
(*7*)	47	F	R hand tenosynovitis and osteomyelitis	None	None	1	E, I, R	Yes	?
(*8*)	32	M	L hand tenosynovitis	None	Local steroid injection	1	E, R, sulfamethoxazole	Yes	24
(*8*)	42	M	L hand tenosynovitis	Shrimp fishing	None	3	E, I, R	Yes	24
(*8*)	52	F	R hand tenosynovitis	Shrimp spine injury	None	1	D, E, R	No	24
(*8*)	31	F	L index finger tenosynovitis	Crab bite	Local steroid injection	6	D, E, R	Yes	24
(*9*)	56	M	L index finger osteomyelitis and wrist synovitis	Fisherman	Local steroid injections	12	E, M, R	Yes	9
(*10*)	5	M	Polyarthritis, disseminated skin lesions, hepatic function abnormalities	None	Abnormalities in monocyte function	48	A, clofazamine, E, R, T	No	9
(*11*)	35	F	L hand and wrist septic arthritis, cutaenous lesions on arm	Puffer fish sting	Systemic steroids for SLE flares	18	M	No	>12
(*12*)	62	F	L middle finger osteomyelitis with skin nodules	Injury while gardening, owned tropical fish aquarium	None	8	E, R	Yes	4
(*13*)	33	M	R hand nodules, LAD, pneumonia, bacteremia	Fish tank	AIDS (CD4<5)	0.5	C, clofazimine, ethionamide	Yes	3 until death
(*14*)	56	M	L ring finger septic arthritis, osteomyelitis	Minor trauma, fish tank	Local steroid injection	2	C, E, R	No	12
(*15*)	0.25	M	Diffuse pustules, osteomyelitis, bacteremia	Bathed in bathtub in which fishtank washed	SCID	?	I, R, T	No	6 until death
(*16*)	71	M	R 2^nd^ MCP synovitis, septic arthritis, osteomyelitis	Swimming and fishing injury	Local steroid injection	9	C, E, I, R	Yes	12
(*17*)	48	F	L hand osteomyelitis, R forearm cutaneous lesions, R ankle septic arthritis and osteomyelitis	Fish tank cleaning	Cyclosphosphamide and systemic steroids for polymyositis	10	D, I pyrazinamide, R	Yes	5
(*18*)	52	F	R hand tenosynovitis and osteomyelitis	Fish dealer, puncture injury	Systemic steroids and local herbal injections	2	Clarithromycin, D, E, I, R	Yes	18
(*19*)	41	F	R index flexor sheath tenosynovitis	Fishmonger	Local steroid injection	3	M	Yes	1.5
(*20*)	53	M	R middle finger tenosynovitis, septic arthritis, wrist osteomyelitis, discharging sinuses	Nonpenetrating trauma, home aquarium	Local steroid injections, systemic methotrexate	24	C, E, R, S	No	13
(*21*)	81	M	L forearm plaque, L index finger ulcer, R forearm cellulitis, bone marro cx + pancytopenia	Aquarium exposure	Systemic steroids and azathioprine for myasthenia gravis	5	C, D	No	<1 before death
(*22*)	22	M	R wrist tenosynovitis, nodular skin lesions	“Fish-related hobby”, aquarium	Systemic steroids for Still’s disease	0.25	C, clarithromycin, E, R	Yes	4
(*23*)	70	M	R hand and wrist tenosynovitis, subcutaneous nodules, L knee septic arthritis	Fish fin wound	Systemic steroids	12	A, E, M, R	Yes	12
(*24*)	60	F	Disseminated ulcerating abscesses	Tropical aquarium	Systemic steroids and chemotherapy for non-Hodgkin lymphoma	8	clarithromycin, clotrimazole, E, immunoglobulins, levofloxacin, R, S,	No	?

^a^Dx, diagnosis; l, left; R, right; MCP,metacarpophalangeal; A, amikacin; C, ciprofloxacin; D, doxycycline; E, ethambutol; I, isoniazid; M, minocycline; P, pyrazinamide; R, rifampin; S, streptomycin; T, trimethoprim/sulfamethoxazole

Most cases occurred in previously healthy adults. The average age was 43 years; 24 (69%) were men; 21 (60%) had tenosynovitis; 6 (17%) had septic arthritis; and 13 (37%) had osteomyelitis. In three patients (9%), either a bone marrow or blood culture positive for *M. marinum* was obtained; all three patients showed marked systemic immunocompromise. Multiple skin lesions were seen in 23% of cases; half of these patients showed clear evidence of deeper infection. Some patients had more than one manifestation of invasive disease. Immunologic impairment was a frequent component of invasive *M. marinum* infections: 14 (40%) of case-patients received a steroid injection at the site of infection, and 9 (26%) were receiving systemic steroids for various indications. An additional 4 (11%) case-patients were in an immunocompromised state from other sources such as chemotherapy or AIDS. Delayed diagnosis was also a prominent finding: The average time to diagnosis was 17 months from symptom onset. The treatment course was prolonged and aggressive: The average treatment duration was 11.4 months in the 20 reports in which a definitive duration was given. Surgery was undertaken in 69% of the cases. The treatment regimen used varied considerably, although 30 (88%) of the 34 patients who took antimycobacterial medications received combination therapy. Rifampin (76%) and ethambutol (68%) were the predominant agents.

While *M.yocobacterium marinum* infections usually arise from aquatic trauma in healthy hosts, delayed diagnosis and immune suppression contribute to the pathogenesis of invasive infection. Tenosynovitis is the most common manifestation of deep invasion, although septic arthritis and osteomyelitis are well described. Disseminated skin lesions can accompany deeper invasion but may be seen in isolation as well. Bone marrow invasion and bacteremia are rare and have been seen only in profoundly immunocompromised patients.

Although the rarity of the condition makes estimating its incidence difficult, the number of case reports per year has remained stable for the last 30 years. However, the high frequency of delayed diagnosis in cases of invasive *M. marinum* disease underscores the importance of maintaining a high level of suspicion for this condition, especially in patients who have evidence of previous aquatic trauma or refractory soft tissue infections. Further, since immunosupression was common in cases of invasive disease, local steroid injections should be avoided in patients with soft tissue infection after aquatic trauma at least until *M. marinum* infection is ruled out by acid-fast staining or mycobacterial culture of biopsy specimens or fluids.

Once invasive *M. marinum* disease was diagnosed, patients with invasive disease were treated for an average of 11.4 months, three times longer than the typical course for *M. marinum* superficial infections (*1*). Rifampin and ethambutol were used most often in invasive infections, although many therapeutic choices exist. In a study of 61 clinical isolates, rifamycins and clarithromycin were the most potent, with the lowest MICs, and resistance was uncommon. Doxycycline, ethambutol, and minocycline all showed higher MICs but were still effective (*1*). A different group tested 11 agents against 37 clinical isolates and found that trimethoprim/sulfamethoxazole was the most potent agent, but 92% of isolates were susceptible. Clarithromycin and minocycline, by contrast, showed susceptibility rates approaching 100% and retained similar potency (*25*). This study reported an MIC_50_ for most quinolones of 4 μg/mL or higher, although in a different study, 100% of *M. marinum* isolates were susceptible to gatifloxacin (*26*). Approximately three fourths of isolates in this latter study were susceptible to ciprofloxacin and levofloxacin. Among newer antibiotics tested against *M. marinum* in this series, only linezolid showed much promise (*26*). On the basis of the sparse data correlating susceptibility testing results to clinical response, and the relative infrequency of resistance, recent guidelines suggest foregoing susceptibility testing in *M. marinum* infections unless the infection does not respond to treatment (*27*). Most cases of invasive *M. marinum* infection require surgical debridement, 69% in this series. This approach seems particularly appropriate in immunocompromised patients, those with tenosynovitis, or those for whom medical therapy fails.
